# Effect of pestle needle combined with six healing sounds Qi Gong on inflammatory factors in patients with lumbar disc herniation

**DOI:** 10.5937/jomb0-55195

**Published:** 2025-06-13

**Authors:** Junzhu Chen, Xiaohui Huang, Chaofan Liu, Yue Feng, Shurong Zhang, Qi Wang, Fang Wang

**Affiliations:** 1 Chengdu University of Traditional Chinese Medicine, Department of Nursing, Chengdu, China; 2 Guang'an Hospital of Traditional Chinese Medicine, Department of Anorectal, Guang'an, China; 3 Guang'an Hospital of Traditional Chinese Medicine, Dean's office, Guang'an, China

**Keywords:** pestle needle, six healing sounds Qi Gong, lumbar disc herniation, inflammatory factors, igla tučka, šest isceljujućih zvukova Ki Gong, lumbalna diskus hernija, inflamatorni faktori

## Abstract

**Background:**

To evaluate the impact of combining Pestle needle therapy with Six Healing Sounds Qi Gong exercises on serum inflammatory markers in patients with lumbar disc herniation.

**Methods:**

Ninety-six patients with lumbar disc herniation (LDH) were randomly assigned to three groups with 32 patients in each group. Group A received standard LDH treatment and care, while Group B received Pestle needle therapy in addition to the standard treatment of Group A. Group C incorporated the Six Healing Sounds Qi Gong in addition to the treatments in Group B. All three groups underwent treatment for four weeks. Comparisons of serum levels of IL-1b, IL-6, TNF-a, and CRP, as well as evaluations using the VAS, the ODI, and JOA score, were conducted among the groups before and after treatment.

**Results:**

Following treatment, the levels of serum inflammatory markers, as well as VAS score and ODI score, in both Group B and Group C, were significantly reduced compared to their pre-treatment levels (P < 0.05). Additionally, the JOA score in these groups was significantly increased compared to pre-treatment values (P < 0.05). These improvements were notably greater than those observed in Group A (P < 0.05). Except for IL-1b, the improvements in the other indices in Group C were significantly better than those in Group B (P < 0.05).

**Conclusions:**

The combination of pestle needle and Six Healing Sounds Qi Gong exercise therapy can effectively reduce serum levels of serum inflammatory markers in patients with LDH. This approach helps alleviate lumbar pain and improve lumbar function.

## Abstract (sr)

**Uvod:**

Za procenu uticaja kombinovana je terapija iglom pestom sa vežbama Qi Gong sa šest zvukova isceljenja na serumske inflamatorne markere kod pacijenata sa hernijom lumbalnog diska.

**Metode:**

Devedeset šest pacijenata sa hernijom lumbalnog diska (LDH) nasumično je raspoređeno u tri grupe sa po 32 pacijenta u svakoj grupi. Grupa A je primala standardni LDH tretman i negu, dok je Grupa B primala terapiju iglom pestom pored standardnog tretmana Grupe A. Grupa C je uključila šest zvukova isceljenja Ki Gong kao dodatak tretmanima u Grupi B. Sve tri grupe su bile podvrgnute tretmanu za četiri nedelje. Poređenja serumskih nivoa IL-1b, IL-6, TNF-a i CRP, kao i evaluacije korišćenjem VAS, ODI i JOA skora, sprovedene su među grupama pre i posle tretmana.

**Rezultati:**

Nakon tretmana, nivoi serumskih inflamatornih markera, kao i VAS skor i ODI skor, u Grupi B i Grupi C, značajno su smanjeni u poređenju sa nivoima pre tretmana (P < 0,05). Pored toga, JOA skor u ovim grupama je značajno povećan u poređenju sa vrednostima pre tretmana (P < 0,05). Ova poboljšanja su bila znatno veća od onih uočenih u Grupi A (P <0,05). Osim za IL-1b, poboljšanja ostalih indeksa u grupi C bila su značajno bolja od onih u grupi B (P < 0,05).

**Zaključak:**

Kombinacija igle tučka i terapije vežbom sa šest zvukova isceljenja Ki Gong može efikasno smanjiti nivoe serumskih inflamatornih markera kod pacijenata sa LDH. Ovaj pristup pomaže u ublažavanju lumbalnog bola i poboljšanju lumbalne funkcije.

## Introduction

Lumbar Disc Herniation (LDH) is a prevalent condition encountered in spinal surgery, characterized by the displacement of lumbar intervertebral disc tissue beyond its normal boundary. This can result in clinical symptoms such as weakness, numbness, pain, and dysfunction in areas innervated by the affected nerves [1]. The global incidence of LDH is approximately 20%–30% [2], profoundly impacting patients’ quality of life and potentially leading to motor dysfunction and even loss of self-care abilities, thereby placing a significant burden on healthcare systems [1] [2] [3].

Research indicates that inflammation is a primary cause of sciatica in LDH patients. The nucleus pulposus of the herniated disc acts as an autoantigen, triggering an autoimmune response that generates numerous immune and inflammatory factors [4], including interleukin-1β (IL-1β), interleukin-6 (IL-6), and tumor necrosis factor (TNF-α) [5] Elevated levels of C-reactive protein (CRP) have been positively correlated with pain and disc degeneration [6].

Current guidelines for managing low back pain recommend combining exercise therapy with physical therapy to alleviate symptoms and enhance patients’ quality of life [3]. Acupuncture, when combined with traditional exercise approaches, has shown effectiveness in treating low back pain and has been confirmed as beneficial for LDH in previous studies. Pestle needle therapy provides multi-target and multilevel effects typical of acupuncture in treating neuropathy. It also incorporates the benefits of massage without penetrating the skin, increasing the pain threshold of local tissues, promoting significant vasodilation, and enhancing the absorption of inflammatory exudates to alleviate pain.

Six Healing Sounds Qi Gong is a classic form of respiratory daoyin exercise that can regulate spinal function and maintain spinal stability by strengthening respiratory muscles, which benefits the rehabilitation of LDH patients [7]. This study explores the mechanisms by which pestle needle therapy combined with Six Healing Sounds Qi Gong exercise may benefit LDH treatment, focusing on the inflammatory response aspects.

## Materials and methods

### Ethics statement

This study involving humans complied with the Declaration of Helsinki and was approved by the Medical Ethics Committee of Guang’an Hospital of Traditional Chinese Medicine and informed consents were obtained from each human donors or the guardians. The number of approval was 2024KL-03.

### Clinical data

A total of 96 hospitalized patients with Lumbar Disc Herniation (LDH), who were treated conservatively in the Orthopedics Department at Guang’an Hospital of Traditional Chinese Medicine from January 2024 to October 2024, were included in this study. Using a random number table method, the patients were divided into three groups: a control group (Group A, n = 32), a Pestle Needle Group (Group B, n = 32), and a Pestle Needle combined with Six Healing Sounds Qi Gong Exercise Group (Group C, n = 32).

### Inclusion Criteria:

A confirmed diagnosis of lumbar disc herniation.

Aged between 18 and 65 years, with no history of analgesic drug treatment in the past month.

Patients with good compliance, having signed the study notice with informed consent.

Participants meeting all the aforementioned criteria were eligible for inclusion.

### Exclusion Criteria:

Presence of lumbar tumor, tuberculosis, spondylolisthesis, severe osteoporosis, a massive protrusion of the lumbar intervertebral disc, excessive spinal canal stenosis, or other surgical indications accompanied by severe neurological dysfunction.

Patients with physical activity disorders.

Patients with skin damage on the waist and back that precludes treatment.

Patients with serious diseases of the heart, brain, liver, kidneys, endocrine system, hematopoietic system, or mental disorders that impede participation in the study.

Individuals with a history of cancer, tuberculosis, hepatitis, or AIDS.

Patients undergoing TCM interventions outside of conventional TCM treatment.

Patients with contraindications to study drugs or a history of allergies.

Patients participating in another clinical trial concurrently.

Pregnant women.

Any participants meeting these exclusion criteria were omitted from the study. Ultimately, 96 LDH patients completed the study.

### Blinded design and implementation

This study adopted a randomized controlled trial design. Due to the specificity of the interventions, it was not possible to implement blinding for the treatment implementers or the patients. Therefore, blinding was applied to the outcome assessors and data analysts, who were not involved in the treatment and were unaware of the specific group assignments. Additionally, the roles of the treatment implementers, outcome assessors, and data analysts were kept separate.

### Treatment methods

### Control group (Group A)

In accordance with the physician’s recommendations, patients received an intravenous administration of famotidine (20 mg) and Compound Vitamin B 1CO once daily. Additionally, weekly health education sessions were conducted by study team members. These sessions covered topics such as dietary guidelines for managing LDH, exercises for strengthening lumbar and back muscles, medication management, and strategies for preventing and treating complications.

### Pestle needle group (Group B)

Building upon the treatment regimen for Group A, Pestle Needle Therapy was introduced. This therapy utilized the Taiji Pestle Needle in accordance with the principles outlined in the Pestle Needle Science manual [8]. The primary acupoints selected for this treatment included: the Eight Formations of the Mingmen Point (GV4), the Eight Formations of the Yaoyangguan Point (GV3), Heche Mingqiangduan, Weizhong Point (BL40), Chengshan Point (BL57), Huantiao Point (GB30), and Zhibian Point (GB32).

The »Eight Formations of the Mingmen Point (GV4)« refers to a technique where the GV4 acts as the central point, located in the depression below the spinous process of the second lumbar vertebra. This technique involves drawing a circle with the distance to the left and right Zhishi points (BL23) as its radius, which is then divided into eight equal sections, all aligned on a straight line. The distance from the center to the perimeter is further divided into three equal segments, forming three concentric circles. Finally, lines are drawn from the center to the eight points on the outer circle, creating the Inner Eight Trigrams, Middle Eight Trigrams, and Outer Eight Trigrams.

The »Eight Formations of the Yaoyangguan Point (GV3)« refers to a method where the GV3 serves as the center, located in the depression below the spinous process of the fourth lumbar vertebra and level with the anterior superior iliac spine. A circle is drawn using the distance to the Dachangshu Point (BL25) as its radius, establishing the points for the Eight Trigrams.

The »Heche Mingqiangduan« refers to the midline extending from the GV4 to the Changqiang Point (GV1), along with three parallel lines on either side of this midline. The first line is positioned 0.5 cun (approximately 1.5 cm) lateral to the Governing Vessel (the spine). The second line is located 1.5 cun (approximately 5 cm) lateral to the Governing Vessel and does not coincide with the first line of the Bladder Meridian of the Foot Taiyang. The third line is situated 3 cun (approximately 10 cm) lateral to the Governing Vessel, aligning with the second line of the Bladder Meridian of the Foot Taiyang.

The GV4 and GV3 Eight Arrays techniques are akin to an even method of tonification and sedation. This technique involves using the tip of a Kui Xing (Star of Ku) pestle to repeatedly tap and apply a specific point-tapping method. Additionally, a Wuxing Santai pestle is utilized to create a Tai Chi motion, moving from the center to the outer edge of the Eight Arrays points in a circular, clockwise manner. Finally, a Vajra pestle is employed to exert penetrating pressure: it is applied vertically downward against the skin for “opening” and raised without losing contact with the skin for “closing.” Each technique is executed for 7 repetitions per cycle, with a total of four cycles.

The Heche Mingqiang technique involves using the tip of a Kui Xing (Star of Ku) pestle to perform repeated tapping, employing a point-tapping method. This is followed by the use of a Vajra pestle to carry out opening and closing movements. Next, the Qiyao Hunyuan pestle is applied along the Heche pathway to execute the separating technique, pushing laterally to create separation and then vertically to align. This is complemented by an ascending and descending motion to perform the lifting technique. Each technique is repeated 7 times per cycle, with a total of four cycles.

The techniques at BL40, BL57, GB30, and GB32 are performed in sequence, utilizing point-tapping and opening-and-closing methods. Each technique is repeated 7 times per cycle, with a total of four cycles.

The relevant examinations were completed on the day of admission (the first day of hospitalization), and the Pestle Needle group received their first Pestle Needle intervention on the second day of hospitalization. The intervention was administered once daily, with each session lasting approximately 30 minutes. After every 5 days of intervention, there was a 2-day rest period, and the entire treatment regimen lasted for a total of 4 weeks.

### Pestle needle combined with Six Healing Sounds Qi Gong exercise group (group C)

Building on the treatment protocol for Group B, the Six Healing Sounds Qi Gong exercises are incorporated as follows:

Hush: Inhale through the nose, then exhale through the mouth while producing a hissing sound. During exhalation, the eyes should be wide open and glaring.

He: Cross the fingers of both hands and raise them above the head. Inhale as the hands move upward, then exhale while making the “he” sound. As you exhale, lower the hands back to the head.

Hehe: Inhale through the nose and exhale while making the »hehe« sound. This can be performed while lying down, but if that is not convenient, it can also be done while standing or sitting.

Exhalation: Inhale through the nose, then exhale through a pinched mouth, making an “exhalation” sound.

Xi: Inhale through the nose, slowly raise your hands with palms facing upward. As you exhale, make the »xi« sound while slowly lowering your hands.

Chui: Exhale while making the »chui« sound, squatting with the legs bent. As you stand up, inhale through the nose.

Each exercise should be performed for 30 minutes once a day. After completing one course (5 days of practice), allow a 2-day rest period before starting the next course. The treatment should continue for a total of 4 courses.

### Observation indicators

### Main outcome measures: Blood inflammatory factor levels

A total of 5 mL of non-anticoagulated peripheral venous blood was collected from the patients both before and after 4 weeks of the intervention. The serum samples were separated and stored in EP tubes at -20°C for future analysis. The levels of IL-1β, IL-6, TNF-α, and CRP were measured using an ELISA. The ELISA kits were purchased from Shanghai Enzyme-Linked Biotechnology Co., Ltd.

### Secondary outcome indicators

(1) Degree of Pain: Pain assessment was conducted using the Visual Analog Scale (VAS), which consists of a 100-mm horizontal line divided into ten equal segments. The scale is anchored by »0« at one end, representing no pain, and »10« at the other end, representing the most severe and unbearable pain.

(2) Waist Dysfunction:

Oswestry Disability Index (ODI): The ODI consists of 10 components, including social life, travel, pain level, ability to perform daily tasks, walking, standing, self-care, sitting, sleep disruption, and sexual activity. Each item is scored on a scale of 0 to 5, with a maximum possible score of 50 points. A higher score indicates greater functional impairment.

Japanese Orthopaedic Association (JOA) Score: The JOA score includes four components: subjective symptoms (9 points), clinical signs (6 points), daily activities (14 points), and bladder function. The maximum score is 29 points, which indicates normal lumbar spine function with no limitations, while a score of 0 points indicates severe lumbar spine dysfunction. A lower score reflects more significant dysfunction.

### Statistical methods

Excel software was used to establish the database, and SPSS version 26 was employed for statistical analysis. All data were evaluated using two-sided tests and confidence interval methods, with a significance level set at α = 0.05. A p-value of <0.05 was considered statistically significant. For normally distributed measurement data were presented as the mean ± standard deviation. Categorical data were expressed as frequencies or percentages. Analysis of variance (ANOVA) was used for comparisons of independent groups with normal distribution and homogeneity of variance, while paired t-tests were applied for intra-group comparisons. The Least Significant Difference (LSD) method was used for post-hoc multiple comparisons. For non-normally distributed data, the rank sum test (Mann-Whitney U test) was applied in non-parametric analyses. The Pearson chi-square test was used to assess differences in categorical data.

## Results

### Baseline demographic and clinical characteristics

Ultimately, 96 LDH patients completed the study. There were no significant differences in baseline characteristics among the three groups (P > 0.05). The data are presented in Table 1.

**Table 1:** Baseline demographic and clinical characteristics.

Groups	age	Gender		Duration of disease<br>(month)
		male	female	
Group A (n=32)	47.41±11.33	10 (23.8%)	22 (40.7%)	18.13±11.74
Group B (n=32)	46.91±11.85	16 (38.1%)	16 (29.6%)	18.0±11.33
Group C (n=32)	46.00±9.76	16 (38.1%)	16 (29.6%)	18.53±13.10
F/χ^2^	0.134	3.048		0.013
P	0.875	0.218		0.987

### Comparison of the levels of inflammatory markers (IL-1β, IL-6, TNF-α, and CRP) before and after treatment across the three groups.

**Figure 1:**
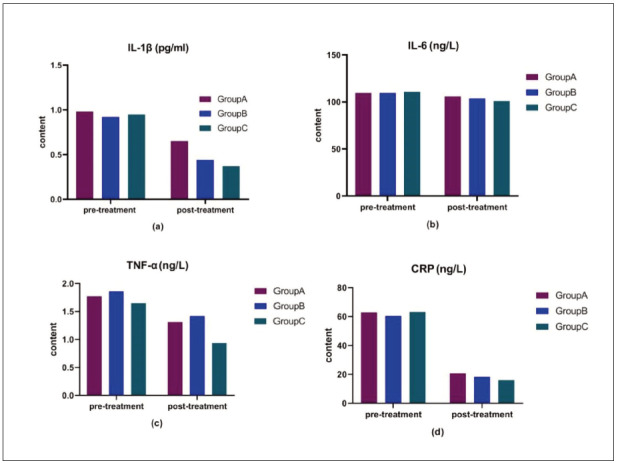
Comparison of serum levels of IL-1β, IL-6, TNF-α and CRP before and after intervention among the three group.

Before treatment, there were no statistically significant differences in the levels of IL-1β, IL-6, TNF-α, and CRP among the three groups (P > 0.05). After treatment, the levels of IL-1β, IL-6, TNF-α, and CRP were significantly lower in all three groups compared to pre-treatment levels (P < 0.05). Additionally, the levels of IL-1β, IL-6, TNF-α, and CRP in groups B and C were significantly lower than those in group A (P < 0.05). The data are presented in Table 2 and Figure 1.

**Table 2:** Comparison of IL-1β, IL-6, TNF-α, and CRP before and after treatment in the three groups. Note: * indicates P<0.05 compared with before intervention in the same group; & indicates P<0.05 compared with group A at the same time point; # indicates P<0.05 compared with group B at the same time point.

Groups	IL-1β (pg/mL)		IL-6 (ng/L)		TNF alpha (ng/L)		CRP (ng/L)	
	Pretreatment	Posttreatment	Pretreatment	Posttreatment	Pretreatment	Posttreatment	Pretreatment	Posttreatment
Group A<br>(n=32)	0.98±0.38	0.65±0.24^*^	109.50±4.87	105.97±5.73^*^	1.77±0.46	1.31±0.46^*^	62.91±10.74	20.76±5.84^*^
Group B<br>(n=32)	0.92±0.23	0.44±0.17^*&^	109.53±6.12	103.68±4.93^*&^	1.86±0.62	1.42±0.51^*^	60.42±15.66	18.32±4.58^*&^
Group C<br>(n=32)	0.95±0.36	0.37±0.19^*&^	110.68±4.99	101.00±4.11^*&#^	1.65±0.41	0.94±0.44^*&#^	63.20±14.21	15.96±3.33^*&#^
F	0.266	16.850	0.512	8.040	1.372	8.956	0.399	8.374
P	0.767	<0.001	0.601	0.001	0.259	<0.001	0.672	<0.001

### Comparison of VAS, ODI, and JOA scores between the two groups before and after treatment

**Figure 2:**
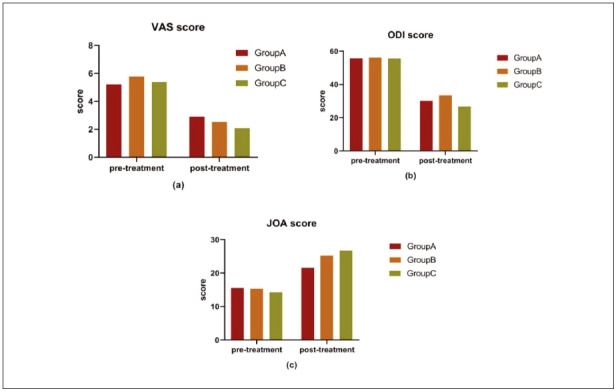
Comparison of VAS score, ODI score and JOA score among the three groups before and after intervention.

Before treatment, there were no statistically significant differences in VAS, ODI, or JOA scoresamong the three groups (P > 0.05). After treatment, the VAS, ODI, and JOA scores were significantly lower in all three groups compared to pre-treatment levels (P < 0.05). Furthermore, the VAS, ODI, and JOA scores in groups B and C were significantly lower than those in group A (P < 0.05). The data are presented in Table 3 and Figure 2.

**Table 3:** Comparison of VAS, ODI and JOA scores before and after treatment in the three groups (scores). Note: * indicates P<0.05 compared with before intervention in the same group; & indicates P<0.05 compared with group A at the same time point; # indicates P<0.05 compared with group B at the same time point

Groups	VAS score		ODI score		JOA score	
	Pretreatment	Posttreatment	Pretreatment	Posttreatment	Pretreatment	Posttreatment
Group A<br>(n=32)	5.22±2.37	2.91±1.00^*^	55.60±4.40	30.17±2.68^*^	15.50±2.37	21.53±1.44^*^
Group B<br>(n=32)	5.78±1.56	2.53±0.51^*^	56.16±5.65	33.43±2.38^*&^	15.28±2.28	25.19±1.82*&
Group C<br>(n=32)	5.38±1.26	2.09±0.96^*&#^	55.58±4.46	26.69±3.27^*&#^	14.22±3.19	26.72±1.28^*&#^
F	0.841	7.301	0.148	46.348	2.151	97.284
P	0.435	0.001	0.863	<0.001	0.122	<0.001

## Discussion

LDH is caused by the extrusion of a degenerative intervertebral disc due to external forces, resulting in the protrusion of the nucleus pulposus, annulus fibrosus, and other disc tissues either outward or posteriorly. In some cases, the annulus fibrosus ruptures, allowing the nucleus pulposus to herniate posteriorly or laterally, which leads to varying degrees of stimulation and compression of the spinal nerve roots. This, in turn, causes symptoms such as numbness and pain in the saddle area, lower back, or lower limbs. LDH most commonly occurs in individuals aged 30 to 50 years [9]. The immune-inflammatory theory of LDH pathogenesis suggests that local inflammation invades the microenvironment of the intervertebral disc (IVD). As the nucleus pulposus (NP) protrudes beyond the IVD boundary due to the destruction of the annulus fibrosus (AF), new nerve fibers and blood vessels abnormally grow into the NP. Ectopic neovascularization and the accompanying inflammatory cells release inflammatory factors, although the precise mechanisms of LDH pathogenesis remain unclear. Infiltration of the inflammatory surface leads to an imbalance in extracellular matrix (ECM) metabolism, cellular senescence, oxidative stress, and abnormal autophagy, ultimately accelerating disc degeneration by promoting endogenous apoptosis and limiting cell proliferation and differentiation [10]. Inflammatory factors and intervertebral disc degeneration are interrelated pathological processes. Inflammatory factors contribute to degeneration by inhibiting ECM synthesis, promoting matrix degradation, and inducing inflammatory responses and apoptosis. Conversely, degenerative disc tissue leads to the upregulation of inflammatory factors such as IL-1β, IL-6, and tumor necrosis factor-α (TNF-α) [11]. Both the Chinese guidelines for the diagnosis and treatment of LDH and the European guidelines for the diagnosis and treatment of neck and low back pain recommend combined treatments for patients with low back pain who are not candidates for surgery. Specifically, they advocate for the combination of exercise therapy with other treatment modalities [4] [12]. Therefore, this study explored the effect of combining Pestle Needle Therapy with Six Healing Sounds Qi Gong on LDH patients by examining changes in inflammatory factors. The results showed that the levels of IL-1β, IL-6, TNF-α, and CRP in Group C and the Group B were significantly lower than those in the Group A (P < 0.05). Notably, the improvement was more pronounced in Group C compared to the other two groups.

Pestle Needle is a special traditional Chinese medicine treatment method that has been passed down for over 60 years within the family of Mr. Li Zhongyu, a renowned expert in acupuncture and moxibustion at the Affiliated Hospital of Chengdu University. It combines the dual characteristics of acupuncture and massage, forming a unique school of acupuncture and moxibustion therapy [13]. In clinical practice, Pestle Needle targets eight specific acupoints and the Heche Road, with additional acupoints added or removed depending on the patient’s condition. These acupoints are primarily located along the Governor Vessel and Bladder Meridian pathways. By stimulating the skin and meridian system in these areas, the treatment regulates the function of the meridians and internal organs (zangfu), promoting blood circulation and unblocking the flow of qi, thereby achieving an analgesic effect. It also facilitates the smooth passage of qi in the Conception Vessel and Governor Vessel, restoring the body’s balance of Yin and Yang, which is considered a state of health in traditional Chinese medicine. Traditional Chinese medicine holds that low back pain, as seen in conditions like LDH, is caused by the blockage of meridians in the waist region, leading to the stagnation of qi and blood. Pestle Needle therapy can effectively unblock these meridians, restoring the flow of qi and blood, akin to the effects of traditional acupuncture. In a study by Peng et al. [14], it was found that acupuncture combined with needle knife treatment for LDH resulted in significantly reduced levels of inflammatory factors—IL-6, IL-10, TNF-α, and MMP-2—thereby alleviating the inflammatory response. In this study, after treatment with Pestle Needle, the levels of inflammatory factors in Group B were significantly lower than those in Group A (P < 0.05), mirroring the results of Peng et al.’s findings. This suggests that Pestle Needle can reduce inflammatory factors in patients with LDH, similar to acupuncture. The therapeutic range of Pestle Needle is broader than traditional acupuncture, and its effects are stable and long-lasting. Pestle Needle therapy combines the benefits of both acupuncture and massage but does not penetrate the skin or muscles. In comparison to traditional acupuncture and moxibustion, Pestle Needle therapy poses no risk of infection, causes less pain for patients, and is more widely accepted. However, some researchers have also applied the Pestle Needle technique to patients with LDH and found it to be effective in improving pain levels and lumbar function [15] [16]. In this study, the VAS and ODI scores in Groups B and C significantly decreased (P < 0.05), while the JOA score significantly increased (P < 0.05) after treatment. These results were significantly better than those in the Group A (P < 0.05), supporting the conclusion that Pestle Needle combined with Six Healing Sounds Qi Gong exercise can significantly improve the symptoms of low back pain and lumbar spine function in patients with LDH.

The Six Healing Sounds Qi Gong, also known as the Six-character Qi formula, is a traditional health cultivation method that primarily utilizes breathing exercises [17]. In this practice, the participant exhales while silently pronouncing six specific sounds—hissing, he, Hehe, exhalation, and Chui—which are believed to regulate the internal organs, thereby contributing to disease prevention and improving overall physical health. From the perspective of traditional Chinese medicine, the Six Healing Sounds Qi Gong method aims to open and balance the corresponding meridians during each exercise. For example, the practice is thought to help unblock energy (Qi) and blood circulation, particularly in the lumbar region, which may benefit patients with LDH by restoring lumbar function and alleviating pain. From a modern medical standpoint, low back pain often results from impaired spinal stability, contraction of the joints, and weakness in the erector spinae and abdominal muscle groups, along with dysfunction in the transversus abdominis muscle. These factors lead to abnormal spinal posture and reduced thoracic mobility, compressing the thoracic cavity and reducing the strength and endurance of the respiratory muscles [18] [19]. Breathing exercises are a crucial component of Six Healing Sounds Qi Gong practice. The diaphragm and abdominal muscles, key components of the respiratory muscle group, form the core muscles, which are essential for maintaining spinal function and promoting limb activity. Core muscle training is effective in protecting the spine, restoring spinal function, and improving the execution of limb movements [2]. Anatomically, the crural diaphragm, part of the diaphragm muscle, attaches in front of the lumbar spine and fuses with the anterior longitudinal ligament. During respiration, the contraction of the diaphragm, transmitted through the crural diaphragm, exerts a stretching effect on the lumbar spine. Additionally, the tension in the abdominal muscles and diaphragm helps maintain intra-abdominal pressure. When the abdominal muscles contract during forced breathing, the increased intra-abdominal pressure supports the spine by sharing the stress with the spinal and lumbar dorsal muscles. This mechanism reduces the load on the spinal extensor muscles, enhances spinal stability, and regulates the curvature of the lumbar spine. Therefore, respiratory movements can positively influence both the morphology and function of the spine through diaphragm contraction and changes in intra-abdominal pressure. In the present study, the VAS score and ODI score of Group C, which participated in Six Healing Sounds Qi Gong exercises, were significantly lower compared to the other two groups (P < 0.05). Moreover, the JOA score for Group C was significantly higher (P < 0.05), indicating that the addition of Six Healing Sounds Qi Gong exercises had a substantial positive effect on reducing low back pain and improving lumbar spine function in patients with LDH. Similar findings were reported by Gordon R and Cortell-Tormo JM [19] [20], who also studied the effects of Six Healing Sounds Qi Gong exercises on LDH patients. Their results showed significant improvements in lumbar dysfunction scores and pain reduction, which align with the present study’s findings The Six Healing Sounds Qi Gong was originally used for the rehabilitation of patients with respiratory diseases. Wang C [21] demonstrated that Six Healing Sounds Qi Gong can significantly reduce levels of inflammatory factors (IL-6 and TNF-α) in patients with chronic obstructive pulmonary disease (COPD), helping to maintain the balance between pro-inflammatory and anti-inflammatory responses and improving the inflammatory response. Li Z [22] also found that Six Healing Sounds Qi Gong can reduce serum levels of inflammatory factors (IL-8 and TNF-α) and increase serum fibronectin (Fn) levels in COPD patients. However, no studies have yet explored the effect of Six Healing Sounds Qi Gong on inflammatory factors in patients with LDH. In the present study, the serum levels of IL-1β, IL-6, TNF-α, and CRP in group C after treatment were significantly lower than those in group A (P < 0.05). Except for IL-1β, the levels of IL-6, TNF-α, and CRP in group C were also significantly lower than those in group B (P < 0.05). These findings suggest that the Six Healing Sounds formula can reduce the levels of inflammatory factors in patients with LDH, relieve pain, and improve lumbar spine function. The lack of a significant difference in IL-1β levels between the present study and the group B may be due to statistical variation or could suggest the involvement of other mechanisms that warrant further exploration.

Our study focused on the improvement of inflammatory markers and lumbar spine function in patients with LDH treated with Pestle needle therapy combined with Liuzi Jiong. The results indicated that the combined therapy effectively reduced inflammatory markers and alleviated pain in patients with LDH. However, there are several limitations, such as the small sample size and short study duration, which may impact the accuracy of the findings. Additionally, the evaluation of lumbar spine function was not sufficiently comprehensive. Therefore, in future studies, we aim to increase the sample size, extend the study duration, and incorporate more objective measures, such as lumbar electromyography, to further investigate the improvement of lumbar spine function.

## Conclusions

In conclusion, the combination of Pestle needle therapy and Liuzi Jue health exercises demonstrates a promising therapeutic effect for patients with lumbar disc herniation. This approach plays a significant role in reducing inflammatory factors and improving lumbar spine function. It is both a safe and effective treatment modality. Therefore, the integration of Pestle needle therapy and the Six Healing Sounds exercise is highly worthy of further promotion and application.

## Dodatak

### Acknowledgements

We extend our heartfelt gratitude to all those who assisted in the preparation of this manuscript. Special thanks are due to Professor Wang Fang for her invaluable guidance and insightful critiques throughout the research process. We are also grateful to the staff at Guang’an Hospital of Traditional Chinese Medicine clinical laboratory for providing the essential facilities and support needed to carry out our experiments. Additionally, we would like to acknowledge the financial support from the Sichuan nursing society, which was crucial in the completion of this project. Our thanks also go to our colleagues from the Department of Nursing, whose suggestions and encouragement were greatly appreciated. Finally, we appreciate the constructive feedback from the anonymous reviewers, whose comments significantly improved the quality of this paper.

### Trial registration

This study has been registered on the international traditional medicine clinical trial registration platform, the URL of the trial registry is http://itmctr.ccebtcm.org.cn/. The registration submission date is November 12, 2024 and the project is under review. The registration number will be provided immediately after the review is passed.

### Funding

This work was supported by the Sichuan Nursing Society 2023 project (No.H23013).

### Data availability statement

The datasets used and/or analyzed during the current study are available from the corresponding author on reasonable request.

### Conflict of interest statement

All the authors declare that they have no conflict of interest in this work.
